# The Influence of Pregnancy on the Caries of the Teeth

**Published:** 1899-05

**Authors:** S. Bird


ARTICLE XIII.

### THE INFLUENCE OF PREGNANCY ON THE CARIES OF THE TEETH.

DR. S. BIRD.

In a number of tables the author records the results of his careful examinations. The teeth of 200 mothers, being premi secundi and multiparæ, of an average age of 18.8 years, were examined and for comparison the caries frequency of 100 multiparæ of an average age of 17.3 was noted, giving the figures of the following table :

| | AGE. | CARIES. |
|---|---|---|
| Mothers, - | 18.8 | 17.9 present. |
| Non-mothers, - | 17.3 | 16.22 " |

By comparing the various tables, the writer concludes that : The caries of mothers is not more frequent than that of non-mothers. The tables further show that there is no increase in the number of carious teeth with the increase of the number of child-births and consequently no influence of pregnancy upon the frequency of caries. And further : In accordance with the comparative results obtained he denies any direct communication between gravity and caries. Pregnant women suffer occasionally from neuralgia of the teeth and gingivitis. The increased acid condition may cause unpleasant effects, but probably the constant hyperæmia of the breath in gravity and in consequence thereof in the pulps of the teeth may bring about the various forms of toothache, more so if there be caries or chronic pulpitis present. Till now the theory has been favored that during pregnancy the teeth lose a large amount of their calcium salts to help in building up the osseous system of the fœtus, and are, therefore, more easily subjected to caries. This theory is absolute and fallacious. If the organism should be in need of a larger amount of lime-salts, the food-stuffs will satisfy this want by an increased assimilation of calcium. Even the poorest food contains more lime than mother and fœtus need. Resorption goes hand in hand with inflammatory symptoms. These might be produced in bone but never in tooth-substance. Furthermore, Williams has shown that formed enamel is not subjected to metabolic changes, and Black's investigations show that the teeth of mothers who have born several times are not any poorer in lime-salts than those of other people.—"Vierteliahresschrift fur Zahnheiikunde."

